# Global Aphasia Secondary to Bilateral Thalamic Hyperintensities Post-cardiac Arrest

**DOI:** 10.7759/cureus.60777

**Published:** 2024-05-21

**Authors:** Kyle N Kaneko, Justin L Hoskin

**Affiliations:** 1 Department of Neurology, Barrow Neurological Institute, Phoenix, USA

**Keywords:** post-cardiac arrest brain injury, diaschisis, thalamic aphasia, thalamus, global aphasia

## Abstract

Thalamic aphasia is thought to occur secondary to disruptions in the cortico-subcortical connections. Although rare, thalamic aphasia is a well-known phenomenon that usually presents with primarily lexical-semantic deficits with preservation of comprehension and repetition. Global aphasia secondary to thalamic injury is extremely rare, with only a few case reports of patients with left thalamic hemorrhages. The prognosis for thalamic aphasia is generally good, with most patients showing little to no symptoms after days or weeks. However, global thalamic aphasia carries a more guarded prognosis with limited recovery months after injury. Here, we report a case of global thalamic aphasia secondary to bilateral thalamic damage post-cardiac arrest.

## Introduction

Thalamic aphasia is a well-known phenomenon that is believed to occur from diaschisis. The thalamus is thought to play a role in language through cortico-subcortical connections. Damage to these connections can disrupt otherwise intact cortical structures, leading to language deficits [1-3]. Thalamic aphasia has been reported in isolated ischemic stroke lesions to the thalamus, mostly occurring in the left anterior region [4]. Aphasia secondary to thalamic hemorrhage has also been reported [5-8]. Aphasia from thalamic lesions can vary but is often mild, with predominately lexical-semantic deficits in speaking and writing and intact repetition and comprehension [1,9]. Recovery from thalamic aphasia is generally quick, usually resolving in days to weeks [9].

Global aphasia is the most severe form of aphasia, with deficits in all functional domains of language. Global aphasia secondary to thalamic lesions is extremely rare, with only a few cases reported in the literature [7,8,10,11]. All cases reported involved the left thalamus and were damaged secondary to intracerebral hemorrhage. To our knowledge, no cases of global aphasia secondary to bilateral thalamic damage from cerebral hypoperfusion without ischemic or hemorrhagic stroke have been reported in the literature. Here, we report the first case of global aphasia secondary to bilateral thalamic hyperintensities caused by cerebral hypoperfusion post-cardiac arrest.

## Case presentation

A right-handed, 69-year-old male with a history of coronary artery disease status post five vessel coronary artery bypass surgery, hyperlipidemia, and hypertension presented after ventricular fibrillation cardiac arrest. He was at home recovering well after his recent coronary artery bypass surgery. He finished eating dinner, went outside to relax, and soon after, his wife found him outside on the ground unresponsive. Emergency medical services were called, and upon arrival, they noted him to be in ventricular fibrillation arrest. Cardiopulmonary resuscitation was initiated; he was defibrillated twice and received three doses of epinephrine. The estimated time to achieve return of spontaneous circulation (ROSC) was about 10 minutes. He was intubated and taken to the nearest hospital. Initial labs were significant for a potassium of 2.4 mmol/L, pH of 7.15, bicarbonate of 16.6 mmol/L, and troponin of 56 ng/mL. Non-contrast head computed tomography (CT) did not show acute intracranial hemorrhage or acute intracranial process. Chest CT angiography was unremarkable, the electrocardiogram (EKG) showed no ST-segment elevation, and the transthoracic echocardiogram (TTE) showed an ejection fraction of 50-55% with no pericardial effusion. There was hypokinesis of the anteroseptal myocardium on TTE, which was consistent with post-operative changes from his recent bypass surgery. Cardiology concluded that his cardiac arrest was secondary to severe hypokalemia and had low suspicion that his graft had occluded given no ST-segment elevation on EKG and a TTE that did not show significant wall motion abnormalities. He initially required pressor support with norepinephrine after ROSC was achieved but was discontinued after improvement in his blood pressure a day after arrival. He required propofol for sedation but was taken off soon after arrival. 

**Figure 1:**
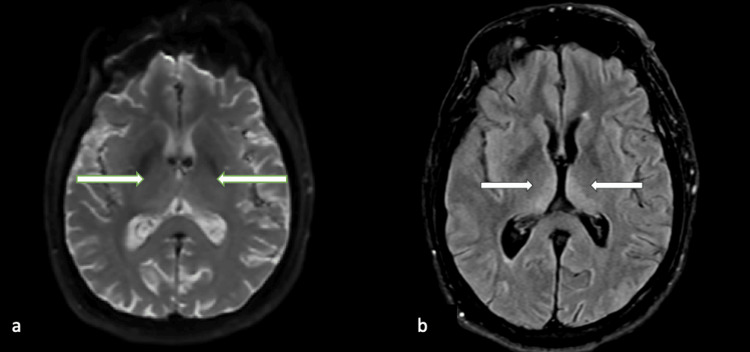
Brain MRI 72 hours post-cardiac arrest a) Diffusion-weighted imaging brain MRI showing no diffusion restriction in bilateral thalami (see arrows). b) Symmetric high FLAIR signal in bilateral medial thalami (indicated by arrows). FLAIR: Fluid-attenuated inversion recovery

On physical exam off sedation, he was intubated with stable vitals. His pupils were symmetric and responsive to light. He was noted to have a left gaze deviation that was overcome with an oculocephalic maneuver. His corneal, cough, and gag reflexes were intact. He moved all extremities spontaneously aside from his right upper extremity. He withdrew to pain in both lower extremities but not in his upper extremities. Given the left gaze deviation, there was concern for seizures or stroke. Continuous video electroencephalogram showed generalized delta activity with triphasic morphology, poorly formed posterior dominant rhythm, and rudimentary sleep architecture, but no seizures. Brain magnetic resonance imaging (MRI) 72 hours post-cardiac arrest showed a symmetric high fluid-attenuated inversion recovery (FLAIR) signal in bilateral medial thalami without restricted diffusion (Figure 1). Magnetic resonance angiography of the head and neck showed no large vessel occlusion or high-grade stenosis. His bilateral thalamic FLAIR hyperintensities explained his decreased level of consciousness and were likely secondary to cerebral hypoperfusion in the setting of recent cardiac arrest. A thiamine level was drawn, and he was empirically started on thiamine supplementation for possible thiamine deficiency as a cause of thalamic FLAIR hyperintensities. His thiamine level returned to the upper limit of normal and thiamine supplementation was discontinued. 

**Figure 2:**
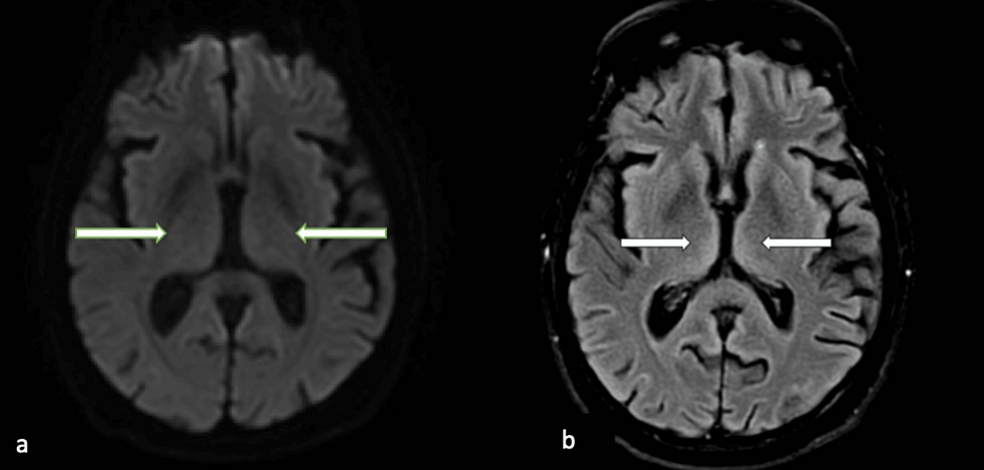
Repeat brain MRI nine days post-cardiac arrest a) Diffusion-weighted imaging brain MRI continued to show no diffusion restriction in bilateral thalami (see arrows). b) Significantly improved FLAIR signal in bilateral medial thalami (indicated by arrows). FLAIR: Fluid-attenuated inversion recovery

On post-cardiac arrest day four, his eyes opened spontaneously, and his gaze deviation resolved, but he was not able to follow commands. On post-cardiac arrest day five, he was extubated and could open his eyes and track but remained unable to follow commands or speak. On post-cardiac arrest day seven, he was able to track well with his eyes but continued to not follow commands or speak. On post-cardiac arrest day nine, a repeat brain MRI with and without contrast was done, given persistent global aphasia despite an improved level of consciousness. Repeat brain MRI showed no acute infarction and prior bilateral thalamic hyperintensities had significantly improved (Figure 2). Despite improvement in his brain MRI, he continued to have global aphasia. One week after his repeat brain MRI, the patient was able to say a few words but continued to be globally aphasic with deficits in fluency, comprehension, and repetition. Two weeks after his cardiac arrest, he was reevaluated and able to speak, but his fluency, repetition, and comprehension were still impaired. Two months after hospitalization, he could talk but could not form complete sentences or speak fluently. His repetition was intact, but his fluency and comprehension remained significantly impaired. He exhibited anomia, unable to name objects like a watch, television, or pen. The patient’s language did not improve over the next six months, and he continued to have significant issues with fluency and comprehension. 

## Discussion

Global aphasia is the most severe form of aphasia, with impairments in all domains of language. Global aphasia usually develops from large lesions affecting both Broca’s and Wernicke’s areas, typically from occlusions to the proximal middle cerebral artery [8]. Rarely aphasia can develop from thalamic lesions through diaschisis and disruption of the cortico-subcortical language network, although not to the degree of global aphasia [9]. Thalamic aphasia is a rare language disorder that generally presents with mild language deficits mostly in naming and semantics, with intact repetition and comprehension [9]. Only a few cases of global aphasia have been reported, all of which involve damage to the left thalamus secondary to hemorrhage [7,8,10,11]. Our case differs from prior case reports of global thalamic aphasia as no hemorrhage or ischemic stroke was seen. Damage to bilateral thalami was seen on T2 FLAIR of our patient’s MRI brain, likely from cerebral hypoperfusion after cardiac arrest leading to vasogenic edema. After cardiac arrest and ROSC, cerebral edema occurs early, and vasogenic rather than cytotoxic edema is seen [12]. This is further supported by negative DWI which suggests no cytotoxic edema. Bilateral thalamic damage explained our patient’s initial decreased level of consciousness. However, even after waking up, he remained globally aphasic. A repeat brain MRI confirmed no damage to his cortical areas, specifically Broca’s and Wernicke’s areas. His repeat brain MRI also showed that his T2 FLAIR thalamic hyperintensities improved significantly, yet his language did not improve, and he remained globally aphasic. The initial injury to the patient’s bilateral thalami likely damaged cortico-subcortical connections involved in language. Despite improvement in vasogenic edema seen on FLAIR imaging, these connections likely remained injured, explaining our patient's persistent language deficits.

In all cases of global aphasia from thalamic damage, the prognosis was guarded, with many patients not recovering deficits months after the injury [7,8]. In contrast, characteristic thalamic aphasia often has a quick recovery, with patients having little to no symptoms after days or weeks [1,9,11]. Our patient had a similar clinical course with prior case reports and did not recover from his language deficits. Although he had some improvement in repetition, his fluency and comprehension remained significantly impaired. 

## Conclusions

Thalamic aphasia usually presents with mild symptoms predominately consisting of lexical-semantic difficulties with preservation of comprehension and repetition. Global thalamic aphasia is extremely rare, with only a few cases reported in the literature. Global thalamic aphasia carries a poor prognosis with a guarded recovery of language. Further research is needed to understand the pathophysiology and risk factors for developing global thalamic aphasia.

## References

[1] Fritsch M, Rangus I, Nolte CH (2022). Thalamic aphasia: a review. Curr Neurol Neurosci Rep.

[2] Stockert A, Hormig-Rauber S, Wawrzyniak M (2023). Involvement of thalamocortical networks in patients with poststroke thalamic aphasia. Neurology.

[3] Maeshima S, Osawa A (2018). Thalamic lesions and aphasia or neglect. Curr Neurol Neurosci Rep.

[4] Fritsch M, Krause T, Klostermann F, Villringer K, Ihrke M, Nolte CH (2020). "Thalamic aphasia" after stroke is associated with left anterior lesion location. J Neurol.

[5] Cappa SF, Vignolo LA, Papagno C, Vallar G (1989). Thalamic aphasia. Neurology.

[6] Alexander MP, LoVerme SR Jr (1980). Aphasia after left hemispheric intracerebral hemorrhage. Neurology.

[7] Ozeren A, Koc F, Demirkiran M, Sönmezler A, Kibar M (2006). Global aphasia due to left thalamic hemorrhage. Neurol India.

[8] Kumar R, Masih AK, Pardo J (1996). Global aphasia due to thalamic hemorrhage: a case report and review of the literature. Arch Phys Med Rehabil.

[9] Rangus I, Fritsch M, Endres M, Udke B, Nolte CH (2022). Frequency and phenotype of thalamic aphasia. J Neurol.

[10] Osawa A, Maeshima S (2016). Aphasia and unilateral spatial neglect due to acute thalamic hemorrhage: clinical correlations and outcomes. Neurol Sci.

[11] Obayashi S (2022). Cognitive and linguistic dysfunction after thalamic stroke and recovery process: possible mechanism. AIMS Neurosci.

[12] Elmer J, Callaway CW (2017). The brain after cardiac arrest. Semin Neurol.

